# MicroRNAs Involved in the Therapeutic Functions of Noni (*Morinda citrifolia* L.) Fruit Juice in the Treatment of Acute Gouty Arthritis in Mice Induced with Monosodium Urate

**DOI:** 10.3390/foods10071638

**Published:** 2021-07-15

**Authors:** Xiaohong Li, Yue Liu, Yaming Shan, Yukun Wang, Zhandong Li, Yingxin Bi, Weihao Zhao, Yuhe Yin, Tianlong Wang, Shuang Li, Fengjie Sun, Changwu Chen, Hao Li

**Affiliations:** 1 College of Food Engineering, Jilin Engineering Normal University, Changchun 130052, China; lixiaohong@jlenu.edu.cn (X.L.); wangyukun5@xdf.cn (Y.W.); lizd591@jlenu.edu.cn (Z.L.); wangtianlong@jlenu.edu.cn (T.W.); chenchangwu@jlenu.edu.cn (C.C.); 2 School of Chemistry and Life Sciences, Changchun University of Technology, Changchun 130012, China; 2202008073@stu.ccut.edu.cn (Y.L.); 2201908056@stu.ccut.edu.cn (Y.B.); 2202008095@stu.ccut.edu.cn (W.Z.); yinyuhe@ccut.edu.cn (Y.Y.); 3 National Engineering Laboratory for AIDS Vaccine, School of Life Sciences, Jilin University, Changchun 130012, China; shanym@jlu.edu.cn (Y.S.); lishuang_19@mails.jlu.edu.cn (S.L.); 4 Key Laboratory for Molecular Enzymology and Engineering, The Ministry of Education, School of Life Sciences, Jilin University, Changchun 130012, China; 5 School of Science and Technology, Georgia Gwinnett College, Lawrenceville, GA 30046, USA

**Keywords:** acute gouty arthritis, mice, monosodium urate, *Morinda citrifolia*, microRNA, noni fruit juice

## Abstract

We investigated the functions of microRNAs in the therapeutic effects of noni (*Morinda citrifolia* L.) fruit juice on mouse models of acute gouty arthritis induced with monosodium urate (MSU). Compared with the model group (treated with MSU), mice in both the positive control group (treated with both MSU and colchicine) and noni fruit juice group (treated with MSU and noni fruit juice) showed a significantly decreased degree of paw swelling in 5 days, as well as the contents of two types of proinflammatory cytokines (i.e., NALP3 and TNF-α). Based on the next-generation sequencing technology, a total of 3896 microRNAs (234 known and 3662 novel) were identified in mice treated with noni fruit juice. A large amount of differentially expressed miRNAs were identified in the noni fruit juice group, suggesting the significant effects of noni fruit juice on the mice with acute gouty arthritis, while the different patterns of change in the numbers of both upregulated and downregulated miRNAs in both noni fruit juice and positive control groups indicated that the mice of acute gouty arthritis may be regulated by differential mechanisms between the treatments of noni fruit juice and colchicine. The target genes of microRNAs involved in the pathogenesis and pathology of acute gouty arthritis in mice were identified and further annotated by both Gene Ontology (GO) and Kyoto Encyclopedia of Genes and Genomes (KEGG) enrichment analyses. Our results revealed the therapeutic effects of noni fruit juice on acute gouty arthritis in mice with a group of microRNAs involved in the pharmacological mechanisms of noni fruit juice, providing scientific evidence to support both the agricultural cultivation and pharmacological significance of noni plants.

## 1. Introduction

Gout is a chronic disease in animals caused by the deposition of monosodium urate (MSU) crystal with typical symptoms of acute, self-limiting inflammatory arthritis affecting the joints in lower limbs [1], showing abnormally high levels of serum uric acids due to the disorders of purine metabolism. Gouty arthritis is one of the most common rheumatic diseases with rising incidence worldwide probably due to the increased incidence of comorbidities and use of causative medications as well as the change of lifestyle, i.e., diet and living habits [2,3]. For example, as recognized as the most common form of inflammatory arthritis, gouty arthritis shows a prevalence of 0.9–2.5% in Europe, 3.9% in the USA, over 6% in some Oceanic–Pacific ethnic groups [4], and 4.5–6.8% in Australia [5], and is predicted with an increasing mortality rate [6], affecting ~3.9% of people worldwide and causing significant clinical burden globally [7,8].

Acute arthritis is recognized as the most common initial symptom of primary gout with typical symptoms including redness, swelling, and severe pain in the affected joints and surrounding tissues [9]. In the occurrence of acute gouty arthritis, the MSU crystals are formed due to the supersaturation of uric acid in the affected joints to interact with local microenvironment, triggering the innate immune response to induce the inflammation cascades and ultimately causing severe inflammation in the joints. In this process, a large number of inflammatory factors and cellular contents are released as key factors involved in the pathogenesis of acute gouty arthritis [10,11]. As a member of the family of the nucleotide-binding oligomerization domain-like receptors (NLRs), cytokine NALP3, also known as nod-like receptor protein 3 (NLRP3), is encoded by gene *NLRP3*, playing an important role in the MSU-mediated gout inflammation and immune regulation [12]. As one of the proinflammatory factors, tumor necrosis factor-α (TNF-α) also plays important roles in the pathogenesis of osteoarticular inflammation, while a large amount of TNF-α is recovered in patients of gout. Molecular studies have shown that the polymorphism in the promoter region of TNF-α gene promotes the development of gouty arthritis [13]. Furthermore, the TNF-α is involved in the expression of pro-IL-1β mRNA and IL-1β protein in gout, triggering the MSU-induced inflammation in mice [14]. Moreover, studies have shown that TNF-α induces the proliferation of synovial fibroblast-like cells and increases the RNA expression in synovial cells, ultimately leading to inflammation [15].

The pathogenetic mechanisms of acute gouty arthritis have recently been widely explored at the molecular, genetic, and genomic levels. The progress in these areas has significantly advanced the identification of potential therapeutic targets in the treatment of acute gouty arthritis with many inflammatory mediators identified as being regulated by microRNAs (miRNAs), which could be potentially used as biomarkers for the diagnosis and prognosis of acute gouty arthritis [16,17]. Furthermore, the promising possibility has been raised to apply genetic testing in personalized treatment of patients with gout [18]. For example, as one of the important proinflammatory cytokines strongly associated with the inflammation of gout, the IL-1β has been identified as the target of several miRNAs [19,20,21,22,23,24], though the regulatory roles of these miRNAs still need to be experimentally verified in vivo [25]. As another proinflammatory cytokine, the TNF-α has been regulated by various types of miRNAs involved in several signaling pathways [19,22,23,24,26,27,28,29,30,31,32,33,34,35,36].

Acute gouty arthritis is generally treated with one or two of the nonsteroidal anti-inflammatory drugs (NSAIDs), colchicine, or corticosteroids, while the xanthine oxidase inhibitor therapy is used to prevent the recurrent gout [1,2,3]. Although these drugs have shown curative effects in a short term, their effectiveness still needs improvement. Furthermore, these common medicines used for a long time in treatment of gout should be taken with precaution due to their severe side effects including gastrointestinal and renal toxicities [37,38,39,40]. Therefore, it is clinically important to develop and identify alternative medicines to treat acute gouty arthritis without toxic and side effects [2]. Notably, traditional Chinese medicines (e.g., extracted agents from some herbal plants) have achieved satisfactory effects in treating gout with low toxicity and reduced side effects [41,42]. Similar effects have been realized by other natural products including curcumin, polydatin, and pterostilbene in treating gout [43,44,45,46]. Several species of plants have been identified as having the anti-gout potential due to their high inhibitory effects on xanthine oxidase [47]. In particular, the effects of fruit or fruit juice intake on treating gout have been complex and controversial [48].

As a member of Rubiaceae and a native tropical shrub in Asia and Polynesia, noni (*Morinda citrifolia* L.) is also known as Indian mulberry in India, mengkudu in Malaysia, painkiller bush in the Caribbean, cheese fruit in Australia, and ba ji tian in China [49]. In the early 1990s, due to its wide applications in health and food industries, the culture of noni plants showed significant contributions in agriculture, industry, and pharmacology, globally spreading from Polynesian and Hawaiian cultures [50,51]. Recently, the complete chloroplast genome of noni was sequenced, providing the foundation for further molecular studies of this economically important plant [52]. Noni fruits have been historically recognized as a traditional medicinal herb for over 2000 years due to their promising medicinal properties [53]. For example, nearly all parts of noni plants (i.e., roots, stem, bark, fruits, and leaves) have been used as curative or preventive treatments of various acute and chronic diseases [54,55], probably functioning by stimulating the immune system [56]. In 2003, Tahitian noni juice was approved as a novel type of food by the Health and Consumer Protection Directorate General of the European Commission [55]. Bioassays in vitro have shown that the molecular mechanisms of the therapeutic effects of the Tahitian noni juice on gout are attributed to its inhibitory effect on the xanthine oxidase [57]. Furthermore, studies have shown that noni fruits contain polyphenolic compounds (e.g., quercetin, rutin, scopoletin, and kaempferol) with potent antioxidant and neuroprotective effects in animals [58]. Noni has also been demonstrated as a novel natural product with prophylactic and therapeutic effects on many human diseases [59] and used as therapeutic agent in antipsychotic, anxiolytic, sedative, and hypnotic treatments [55,60]. Due to the wide spectrum of claimed medical and therapeutic utilities, it is urgent to obtain scientific evidence to verify the pharmacological effectiveness of noni fruit juice. Therefore, it is important to investigate and confirm the therapeutic effects of noni fruit juice and further establish its wide medical applications.

In this study, we investigated the potential therapeutic effects of noni fruit juice on acute gouty arthritis in vivo. We established the mouse model of acute gouty arthritis with the treatment of MSU. Our goals were to evaluate the therapeutic effects of noni fruit juice on acute gouty arthritis in mice by assessing the phenotypic characteristics (i.e., the paw swelling degree in mice), the contents of two types of proinflammatory cytokines (i.e., NALP3 and TNF-α), and the expression patterns of miRNAs, to ultimately illustrate the potential pharmacological mechanisms of noni fruit juice on acute gouty arthritis in mice. We further investigated the potential regulatory roles of miRNAs in the biological processes of the treatment of noni fruit juice on acute gouty arthritis in mice based on both Gene Ontology (GO) and Kyoto Encyclopedia of Genes and Genomes (KEGG) enrichment analyses of the target genes of the differentially expressed miRNAs in mice treated with noni fruit juice.

## 2. Materials and Methods

### 2.1. Experimental Animals

**Figure 1:**
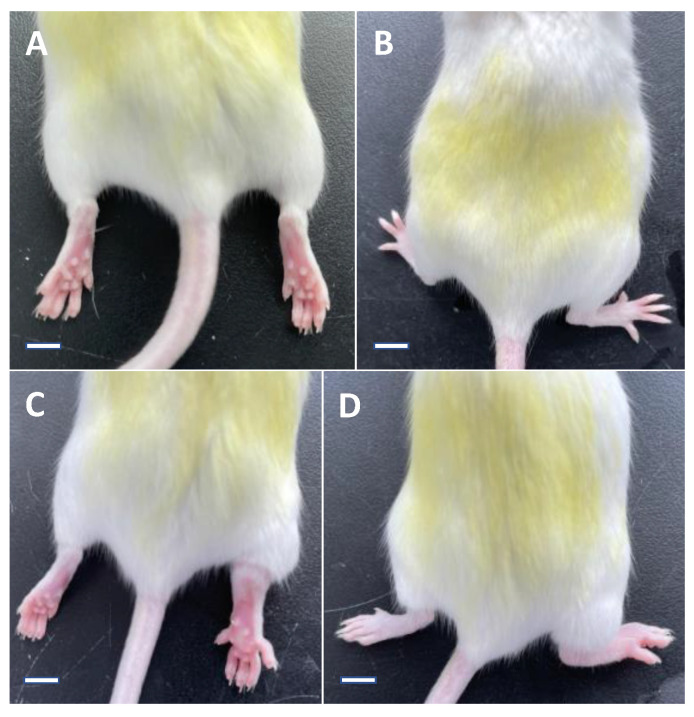
The ventral and dorsal views of the same mouse showing the right hind paw before (**A**,**B**) and 1 h after (**C**,**D**) the application of monosodium urate (MSU). The mice treated with MSU showed evident swelling in their right hind feet, indicating the successful construction of the mouse model of acute gouty arthritis. Bar = 4.15, 5.36, 4.85, and 5.48 mm in (**A**–**D**), respectively.

A total of 40 male Kunming mice (~5 weeks old with an average body weight of 25 ± 3 g) were purchased from the Liaoning Changsheng Biotechnology Co., Ltd. (Shenyang, China) with an animal certificate number of SCXK (Liao) 2015-0001. The selection of this type of mice was based on the previous studies on the acute gouty arthritis in mice [61]. These mice were randomly and evenly divided into four groups, i.e., one normal control group and three experimental groups, including the model group (treated with MSU), the positive control group (treated with both MSU and colchicine), and the noni fruit juice group (treated with both MSU and noni fruit juice). The establishment of the positive control group was based on the results of previous studies showing that the application of colchicine was effective on the treatment of acute gouty arthritis [2,21]. After one week of adaptive feeding, the right hind feet of the 30 mice in the three experimental groups were first disinfected with medical ethanol (75%) and then injected subcutaneously with 0.05 mL of MSU (Sigma, St. Louis, MO, USA) crystal suspension (2.5%) with sterile syringes on both the ventral and dorsal sides of the foot, respectively. In one hour, compared with the 10 untreated mice in the normal control group, the mice treated with MSU showed evident swelling in their right hind feet with no evident liquid overflow, indicating the successful construction of the mouse model of acute gouty arthritis (Figure 1). For the next five days, the mice in the noni fruit juice group and the positive control group were fed with noni fruit juice (7.8 mL/kg d^−1^) and colchicine solution (Sigma, St. Louis, MO, USA) (0.975 mg/kg d^−1^), respectively, while the mice in the model and the normal control groups were fed with saline water (7.8 mL/kg d^−1^). All mice were fed with oral gavage needles. For the entire experiment, the mice were kept in cages with natural sunlight, constant temperature and humidity, and which were regularly cleaned and disinfected. After the experiments, the mice were euthanized with inhalation of carbon dioxide gas.

### 2.2. Production of the Noni Fruit Juice

Fresh and mature noni fruits were obtained from the Fiji Pacific Noni Biotechnology Co., Ltd. (Hainan, China). These fruits were locally picked and kept frozen at −20 °C (Nadi, Fiji). Fruits thawed at room temperature were washed with sterile water, air-dried, cut into blocks each of ~5 mm thickness, and crushed by a fruit crusher. The fruit pulp was heated to ~25 °C, mixed with pectinase (0.225 g/L) while stirring, then inoculated with *Lactobacillus plantarum* (1%) to incubate for 4 h in an incubator (Shanghai Yiheng Scientific Instrument Co., Ltd., Shanghai, China) at 40 °C. Then, the fruit pulp was filtered with the 20-mesh filter cloth and fermented for 20–30 days at 38 °C. The supernatant was collected with a siphon, filtered, sterilized for 20 min at 80–82 °C, and aseptically collected as the fermented noni fruit juice.

### 2.3. Measurement of the Paw Swelling in Mice

To evaluate the effectiveness of noni fruit juice on treating acute gouty arthritis in mice, the phenotypic characteristics were observed by measuring the paw thickness (i.e., the distance between the ventral and dorsal sides of the paw including the foot pad) of mice with a digital vernier caliper before and after (1 and 5 days) the treatments. The paw swelling degree of mice was calculated as the difference between the thickness of the right hind paw before and after (1 and 5 days) the treatments in three experimental and the control groups of mice. Data were presented as mean ± standard deviation and were statistically analyzed using SPSS 19.0. with the significant difference of ANOVA set at *p*-value ≤ 0.05.

### 2.4. Measurement of the Contents of NALP3 and TNF-α

Due to their direct involvement in the pathogenesis of acute gouty arthritis, the proinflammatory cytokines NALP3 and TNF-α were chosen to assess the effectiveness of noni fruit juice on the treatment of acute gouty arthritis in mice. In 5 days, the blood sample (~1.0 mL) was collected from the eyeballs of all mice and incubated in an electrothermal incubator (Shanghai Yiheng Scientific Instrument Co., Ltd., Shanghai, China) for 1 h at 37 °C, kept for 3 h at 4 °C, and centrifuged for 10 min at 3000 rpm to separate the serum (~0.5 mL) from the red blood cells. The supernatant was collected for the enzyme-linked immunosorbent assay (ELISA) analysis to detect the contents of NALP3 and TNF-α using the NALP3 and TNF-α ELISA kits (Shanghai Le Pure Biological Technology Co., Ltd., Shanghai, China), respectively, in accordance with the manufacturer’s instructions with the concentration of the cytokines measured at 450 nm by the microplate enzyme reader (Tecan Austria Gmbh, Salzburg, Austria). Data were presented as mean ± standard deviation and were statistically analyzed using SPSS 19.0. with the significant difference of ANOVA set at *p*-value ≤ 0.05.

### 2.5. High-Throughput Sequencing and Analysis of microRNAs

In order to explore the functions of small RNAs in the pathogenesis and treatment of acute gouty arthritis mediated by MSU and noni fruit juice, respectively, we randomly selected a representative mouse serum sample from each of the four groups of mice to identify the miRNAs using the next-generation sequencing platform BGISEQ-500 (BGI, Shenzhen, China) [62,63]. To extract the total RNA, the QIAzol Lysis Reagent (1000 μL) was added to the serum sample (200 μL), vortexed evenly, and kept for 5 min at room temperature. Then, a total of 200 μL chloroform/isoamyl alcohol (24:1) was added to the reaction, vortexed or shaken vigorously for 15 s, and incubated for 2–3 min at room temperature. The reaction was centrifuged for 8 min at 12,000× *g* and 4 °C (5427R, Eppendorf Co., Ltd., Beijing, China). The supernatant was collected and added with absolute ethanol of twice the volume of the supernatant and mixed well. The mixed solution was filtered through the column for purification, and washed once with buffer RWT (700 μL) and twice with buffer RPE (500 μL). The solution was centrifuged for 2 min at 12,000× *g* and room temperature, and the purification column was moved to a new collection tube with 20 μL RNA-free water, incubated for 1 min at room temperature, and finally centrifuged for 2 min at 12,000× *g* and room temperature to elute RNA. The concentration of the eluted RNA was measured by the Agilent 2100 Bioanalyzer (Agilent Technologies Co., Ltd., Beijing, China). To construct the libraries for high-throughput sequencing of microRNAs, a total of 200–1000 ng of RNA sample was used to filter the small RNAs and to separate RNA segments of different sizes by PAGE gel and the stripes of 18–30 nt (14–30 ssRNA Ladder Marker, TAKARA) were recycled. The connection 3′adaptor system was prepared to complete the adaptor ligation with the reaction condition of 70 °C for 2 min and 25 °C for 2 h; the RT-Primer was added with the reaction conditions of 65 °C for 15 min and ramp to 4 °C at a rate of 0.3 °C/s; the 5′adaptor mix system was added with the reaction condition of 70 °C for 2 min and 25 °C for 1 h. The RT-PCR reaction was conducted as follows: The First Strand Master Mix and Super Script II (Invitrogen) reverse transcription were prepared with the reaction condition of 42 °C for 1 h and 70 °C for 15 min; and several rounds of PCR amplification with PCR Primer Cocktail and PCR Master Mix were performed to enrich the cDNA fragments with the following reaction conditions: 95 °C for 3 min, 15–18 cycles of “98 °C for 20 s, 56 °C for 15 s, and 72 °C for 15 s”, and extension at 72 °C for 10 min; 4 °C hold. The PCR products were purified with PAGE gel and the recycled products were dissolved in EB solution. The double-stranded PCR products were heat denatured and circularized by the splint oligo sequence. The single strand circle DNA (ssCir DNA) was formatted as the final library, which was validated on the Agilent 2100 Bioanalyzer. The library was amplified with phi29 to make DNA nanoball (DNB) containing more than 300 copies of one molecule. The DNBs were loaded into the patterned nanoarray and single-end 50 base reads were generated in the way of combinatorial probe-anchor synthesis (cPAS).

The clean sequencing tags were annotated using Bowtie2 (http://www.bowtie-bio.sourceforge.net/bowtie2/ (accessed on 21 April 2019)) based on various small RNA databases including miRbase (http://www.mirbase.org/ (accessed on 21 April 2019)), Rfam (https://rfam.xfam.org/ (accessed on 21 April 2019)), siRNA (http://web.mit.edu/sirna/ (accessed on 21 April 2019)), piRNA (https://www.pirnadb.org/ (accessed on 21 April 2019)), and snoRNA (http://snoopy.med.miyazaki-u.ac.jp (accessed on 21 April 2019)). Based on the results of these annotations, the unknown tags were further annotated to predict novel miRNAs using miRDeep and miRA [64,65,66].

### 2.6. Identification and Analysis of Differentially Expressed Genes of microRNAs

A stringent algorithm was developed to identify the differentially expressed genes (DEGs) of miRNAs in mice according to Audic and Claverie [67]. By using the false discovery rate (FDR) method, the FDR ≤ 0.001 and the absolute value of Log2Ratio(Fold Change) ≤ 1 were set as the default thresholds to evaluate the significant difference in gene expression. The hierarchical clustering of differentially expressed miRNAs was constructed using the function “pheatmap” of R package (http://www.r-project.org (accessed on 25 April 2019)). The miRNAs were further processed to predict their target genes jointly by both miRanda [68] and TargetScan [69]. The functional enrichment analyses of the target genes of differentially expressed miRNAs were further conducted based on Gene Ontology (GO; http://www.geneontology.org/ (accessed on 25 April 2019)) database using WEGO software [70] and Kyoto Encyclopedia of Genes and Genomes (KEGG; http://www.genome.jp/kegg/ (accessed on 25 April 2019)) database [71], respectively.

## 3. Results and Discussion

### 3.1. Paw Swelling Degree in Mice

The phenotypic characteristics of the mice treated with either colchicine or noni fruit juice showed significant changes in comparison to those of the model group. The results of the paw swelling degree in the mice of these three experimental groups in 1 and 5 days after the treatments were shown in Table 1. In one day, the paw swelling degree in mice of the colchicine group was significantly decreased in comparison to that of the model group, while the significantly decreased paw swelling degree was observed in 5 days in the noni fruit juice group. Overall, the noni fruit juice showed less effectiveness than that of the colchicine in the treatment of swollen paws in mice. These results suggested that the therapeutic effects of the noni fruit juice on acute gouty arthritis started slower with probably a long-term effect in comparison to that of the colchicine [72].

The phenotypic observations of the reduced paw swelling degree in mice are generally used to evaluate the therapeutic effects of medicines on acute gouty arthritis. For example, the ethanol extracts from one of the traditional Chinese medicinal herbal plants (*Gnaphalium pensylvanicum* Willd.) have been shown to reduce the paw swelling in mice likely by decreasing the serum uric acid and inhibiting the xanthine oxidase [73]. Similarly, the combination of ethanol extracts from a few traditional Chinese medicinal herbal plants showed therapeutic effects on acute gouty arthritis by attenuating the degree of ankle swelling and inflammation in mice [9]. These results are consistent with our findings showing the therapeutic effects of noni fruit juice on acute gouty arthritis in mice. The molecular mechanisms of the therapeutic effects of noni fruit juice on acute gouty arthritis in mice were further explored by evaluating the change of the two main proinflammatory cytokines, i.e., NALP3 and TNF-α (below).

### 3.2. Contents of NALP3 and TNF-α

Due to their direct involvement in the pathogenesis of acute gouty arthritis, the levels of both proinflammatory cytokines of NALP3 and TNF-α were detected to evaluate the effectiveness of noni fruit juice on the treatment of acute gouty arthritis in mice. In 5 days after the treatments, the contents of NALPs and TNF-α were presented in Table 2.

In comparison to the model group, the contents of both NALP3 and TNF-α in mice of the normal control, the colchicine, and the noni fruit juice groups were significantly decreased. These findings were in accordance with the molecular pathways of the generation of acute gouty arthritis, showing that upon the recognition of MSU, the molecular structure of NALP3 was altered to activate the NALP3 inflammasome, ultimately generating a large amount of TNF-α [12]. Similar to the results of the treatment of noni fruit juice on the paw swelling degree in mice, these results of the change of both NALP3 and TNF-α in mice demonstrated again the therapeutic effects of noni fruit juice on treating acute gouty arthritis in mice. Overall, the noni fruit juice was less effective than colchicine in reducing the levels of NALPs and TNF-α in mice with acute gouty arthritis. Furthermore, our results were consistent with those previously reported in the treatment of acute gouty arthritis. For example, the expression of both NALP3 and TNF-α, which were involved in the NLRP3/apoptosis-associated speck-like protein/caspase-1 pathways, was significantly decreased in mice of acute gouty arthritis treated with the alcohol extract from *Polygonum cuspidatum* [74]. Similarly, a recent study demonstrated that the chemical extracts from the traditional Chinese medicinal herbal plant (i.e., *Dendrobium loddigesii* Rolfe) showed anti-gout activities in rat model of acute gouty arthritis [75]. Specifically, the chemical extracts inhibited the acute gouty arthritis in rats and showed anti-inflammatory effects as demonstrated by reduced levels of proinflammatory cytokines including IL-1β and TNF-α. Furthermore, studies have shown that the combined ethanol extracts from a few traditional Chinese herbal medicinal plants decreased the levels of TNF-α but not of IL-1β in the rat model of acute gouty arthritis [9]. These results suggested that the noni fruit juice showed therapeutic effects on acute gouty arthritis and that the chemical extracts of different herbal plants may be involved in their therapeutic effects on acute gouty arthritis with different molecular mechanisms. As one of the proinflammatory mediators involved in the pathogenesis of acute gouty arthritis, TNF-α has been constantly shown with increased expression in the subjects of acute gouty arthritis [22]. Notably, the polysaccharides extracted from noni fruits have been shown to decrease the levels of TNF-α in mice treated with acetic acid [49]. These results suggested that the noni fruit juice extracted in our study may contain the same or similar chemical contents as the polysaccharides as reported previously [49,76]. Future studies are necessary to further identify and characterize the functional substances in noni fruit juice [77].

Studies have shown that the proinflammatory cytokines (e.g., IL-1β and TNF-α) are regulated by various types of miRNAs [19,22,37,78,79,80]. Similarly, several miRNAs have been identified as potential markers associated with NALP3 in the development of acute gouty arthritis [17,80]. These results strongly indicated that the noni fruit juice showed its therapeutic effects on acute gouty arthritis by inhibiting the TLR/MyD88/NF-kB pathways (i.e., reducing the content of TNF-α). Further studies are necessary to investigate the mechanism of noni fruit juice in regulating the expression of these cytokines in the occurrence of acute gouty arthritis. We further explored the potential regulatory functions of miRNAs in the treatment of noni fruit juice on mice of acute gouty arthritis (below).

### 3.3. Small RNA Sequencing

#### 3.3.1. Characteristics of Small RNAs

To investigate the therapeutic effects of noni fruit juice on acute gouty arthritis in mice, miRNAs were sequenced using the next-generation sequencing platform BGISEQ-500 technology. The low-quality tags were removed prior to further data analyses (Table 3). The results of the length distribution analysis showed that the composition of the small RNAs generally ranged from 18 to 30 bases in length with the miRNAs of 21–22 bases.

There was a total of 92,262,998 (~82.22%) valid reads (out of 112,216,741 raw tags) obtained in four groups of mice with 84,745,425 clean tags (~91.85%) identified as various types of RNAs (Figure S1; Table S1). The raw sequencing data were deposited in the Sequence Read Archive (SRA) at the National Center for Biotechnology Information (NCBI) (http://www.ncbi.nlm.nih.gov/sra (accessed on 7 April 2021)) under the BioProject accession number of PRJNA719968. In both the noni fruit juice and normal control groups, most small RNAs with higher proportions ranged from 10 to 33 bases with the most abundant small RNAs of 20 and 31 bases. In the model group, the length distributions of most small RNAs ranged from 18 to 22 bases with the most abundant small RNAs of 20 bases, while the length distributions of most small RNAs ranged from 29 to 33 bases with the most abundant small RNAs of 31 bases in the colchicine group. A total of 271, 350, 331, and 234 known miRNAs and a total of 2146, 1396, 581, and 3662 miRNAs were identified as novel in the normal control, the model, the colchicine control, and the noni fruit juice groups, respectively (Table 3 and Table S2).

#### 3.3.2. Differentially Expressed microRNAs

**Figure 2:**
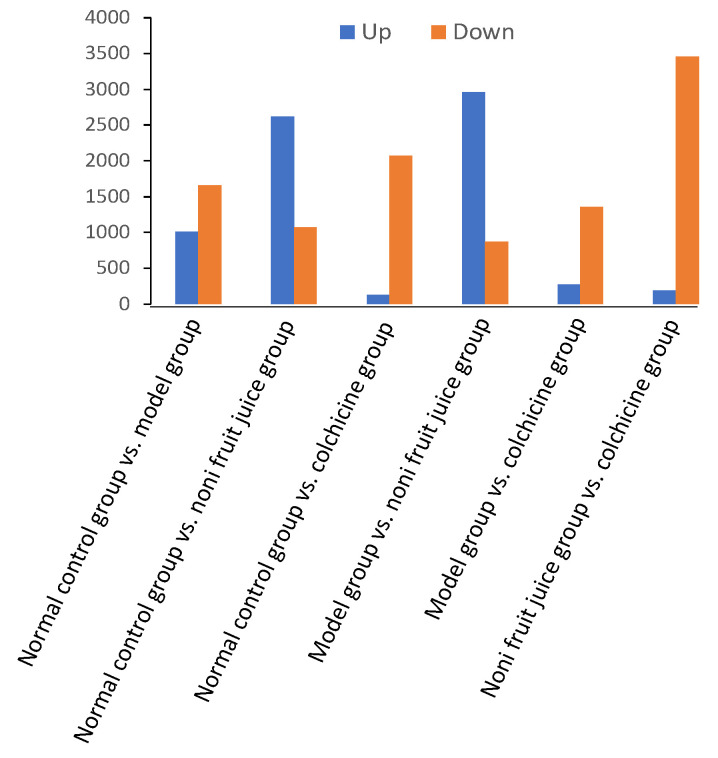
Differentially expressed microRNAs in the six pairwise comparisons of the three experimental and the normal control groups of mice.

Differentially expressed miRNAs (Table S3) were identified based on the values of FDR ≤ 0.001 and |Log2Ratio(Fold Change)| ≤ 1 in the six pairwise comparisons among the three experimental and the control groups of mice in order to evaluate the variation in miRNA gene expression profiles in response to MSU (the model group), colchicine (the positive control group), and noni fruit juice in mice (Figure 2). The heatmaps based on intersection and union of miRNAs in four groups of mice (Figure S2) and differentially expressed miRNAs in six pairwise comparisons of four groups of mice (Figure S3) were generated to show the hierarchical clustering of these miRNAs.

The results showed that the highest numbers of differentially expressed miRNAs were revealed in the noni fruit juice group (Figure 2). Two of the six pairwise comparisons of experimental and control groups, i.e., the normal control group vs. noni fruit juice group and the model group vs. noni fruit juice group showed two of the highest number of upregulated miRNAs of 2624 and 2963, respectively. The highest number of downregulated miRNAs (3460) were revealed in the comparison between the noni fruit juice group vs. colchicine group, while the lowest numbers of upregulated miRNAs of 130, 275, and 191 were revealed in the pairwise groups of the normal control group vs. colchicine group, the model group vs. colchicine group, and the noni fruit juice group vs. colchicine group, respectively. The other three pairwise comparisons (i.e., the normal control group vs. model group, the normal control group vs. colchicine group, and the model group vs. colchicine group) showed relatively smaller numbers of differentially expressed miRNAs of 2670, 2205, and 1635, respectively. Evidently, the large amount of differentially expressed miRNAs were identified in the noni fruit juice groups, indicating the significant effects of noni fruit juice on the mice with acute gouty arthritis. The large number of down-regulated miRNAs suggested the additive effects of both noni fruit juice and colchicine on the mice with acute gouty arthritis, while the different patterns of change in the numbers of both upregulated and downregulated miRNAs in both noni fruit juice and colchicine groups suggested that the mice of acute gouty arthritis may be regulated by differential mechanisms between the treatments of noni fruit juice and colchicine. Further studies are needed to identify the explicit functions of these miRNAs in the development and therapeutic treatment of acute gouty arthritis in mice.

#### 3.3.3. Target Genes of microRNAs in Mice

**Figure 3:**
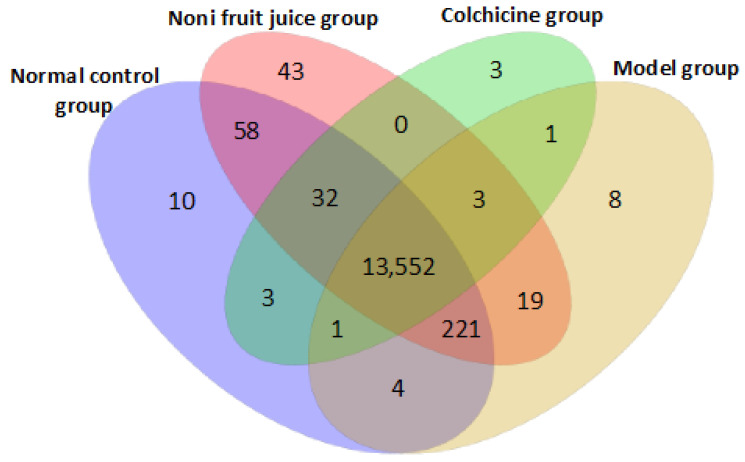
Target genes of microRNAs predicted by both TargetScan and miRanda in four groups of mice.

A total of 13,958 target genes of the miRNAs in the experimental and control groups of mice were predicted based on both miRanda and TargetScan with a total of 13,552 target genes identified in all four groups of mice (Figure 3; Table S4). Results showed that in the pairwise comparisons, a total of 133 and 14 target genes were uniquely identified in the noni fruit juice and the model groups, respectively, while a total of 38 and 252 target genes were exclusively revealed in the colchicine and the model groups, respectively. Furthermore, when the four groups of mice were compared, a total of 10, 43, 3, and 8 target genes annotated by a total of 10 novel and 1 known, 60 novel, 3 known, and 9 novel and 1 known miRNAs were exclusively identified in the normal control, the noni fruit juice, the colchicine, and the model groups of mice, respectively. These results suggested that the therapeutic effects of noni fruit juice on acute gouty arthritis in mice were probably regulated by different molecular mechanisms from those of colchicine. Future studies are necessary to investigate the explicit functions of these miRNAs and their target genes in the pathology and pathogenesis of acute gouty arthritis and the pharmacological mechanisms of noni fruit juice in treating acute gouty arthritis in mice.

#### 3.3.4. Enrichment Analyses of Target Genes of differentially Expressed microRNAs

**Figure 4:**
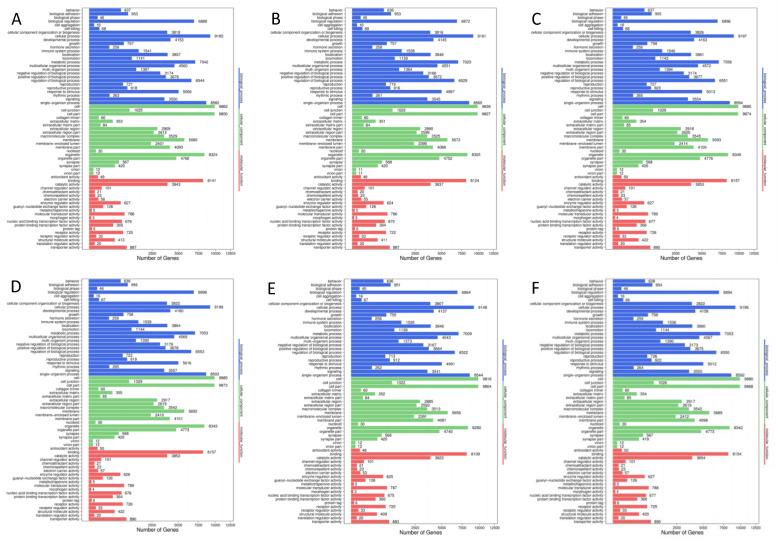
Functional annotations based on the Gene Ontology (GO) database of the differentially expressed microRNAs identified in the six pairwise comparisons of the three experimental and the control groups in mice, including normal control group vs. model group (**A**), normal control group vs. colchicine group (**B**), normal control group vs. noni fruit juice group (**C**), model group vs. noni fruit group (**D**), model group vs. colchicine group (**E**), and noni fruit juice group vs. colchicine group (**F**). Comparable results were revealed among the six pairwise comparisons of the experimental and control groups of mice, showing the largest and the lowest numbers of genes annotated in the categories of cellular component and biological process of the GO database, respectively.

Gene Ontology (GO) enrichment analysis was performed to annotate the functions of target genes of the differentially expressed miRNAs identified in the six pairwise comparisons among the three experimental and the control groups of mice. Results showed that a total of ~78,000 (ranging from 78,445 to 78,864), ~56,000 (ranging from 56,319 to 56,744), and ~16,000 (ranging from 16,841 to 16,953) target genes were annotated in three categories of GO database, i.e., cellular component, molecular function, and biological process, respectively (Figure 4; Table S4). Similar patterns of gene annotation were observed in the six pairwise comparisons of the experimental and control groups in mice, with the greatest number of genes mapped to the category of biological process of GO terms, while the category of molecular function contained the least number of genes annotated. In the category of biological process of GO database, five GO terms showed the highest numbers of functional genes annotated (~6500–9100), including cellular process, single-organism process, metabolic process, biological regulation, and regulation of biological process, while the other 21 terms each contained less than 5200 genes annotated ranging from 16 to 5016. In the category of cellular component in GO database, three GO terms (i.e., cell, cell part, and organelle) showed the highest numbers of functional genes annotated (~8200–9800), while the other 16 terms each contained less than 5700 genes annotated ranging from 11 to 5693. In the category of molecular function, two GO terms (i.e., binding and catalytic activity) were revealed to contain the largest numbers of annotated genes (~3800–8100), while the other 18 terms each contained less than 900 genes annotated ranging from 3 to 890.

As one of the major pathways involved in the pathology of acute gouty arthritis, the formation of NLRP3 (NALP3) inflammasome complex and the regulations of NLRP3 inflammasome complex assembly were further investigated based on the GO annotation of the target genes of differentially expressed miRNAs identified in the six pairwise comparisons of the experimental and control groups of mice (Table 4; Table S5). In the categories of both cellular component and biological process in GO database, many up- and downregulated novel miRNAs were involved in the formation of NLRP3 inflammasome complex and the positive and negative regulations of NLRP3 inflammasome complex assembly. These results indicated that the metabolic pathways of NLRP3 inflammasome complex was affected in mice treated with noni fruit juice, suggesting that these differentially expressed miRNAs of these GO terms may be related to the metabolic pathways of the NLRP3 inflammasome complex. These results were consistent with those based on the pathogenetic investigations in our study and with those reported previously [17]. Further studies are necessary to investigate the detailed regulatory functions of these novel miRNAs in the occurrence of acute gouty arthritis and the pharmacological mechanisms of noni fruit juice in treating acute gouty arthritis in mice.

**Figure 5:**
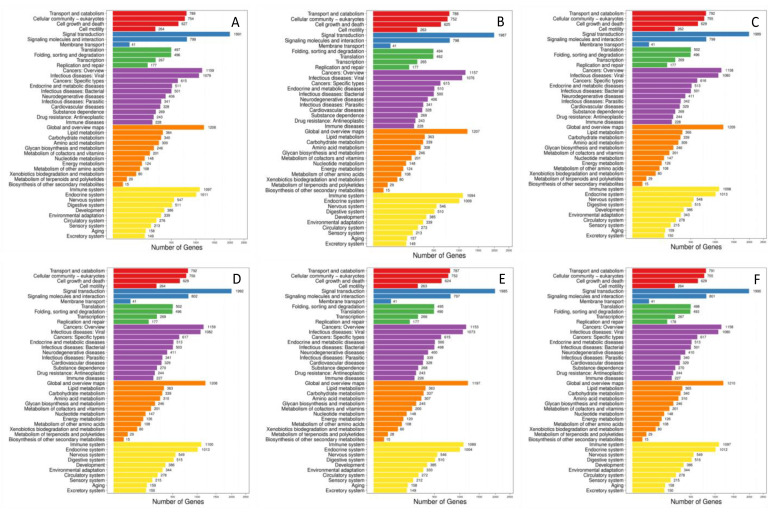
Metabolic pathway enrichment analysis based on the Kyoto Encyclopedia of Genes and Genomes (KEGG) database of the differentially expressed microRNAs identified in the six pairwise comparisons of experimental and control groups of mice, including normal control group vs. model group (**A**), normal control group vs. colchicine group (**B**), normal control group vs. noni fruit juice group (**C**), model group vs. noni fruit group (**D**), model group vs. colchicine group (**E**), and noni fruit juice group vs. colchicine group (**F**). Comparable results were revealed among the six pairwise comparisons of the experimental and control groups of mice, showing the highest and the lowest numbers of differentially expressed miRNAs enriched in the categories of human diseases and genetic information processing of the KEGG database, respectively. The six color blocks (i.e., red, blue, green, purple, orange, and yellow) represent six categories of metabolic pathways, i.e., cellular processes, environmental information processing, genetic information processing, human diseases, metabolism, and organismal systems, annotated by the KEGG database, respectively. Number of genes represents the target genes of the differentially expressed miRNAs.

The metabolic pathway enrichment analysis of the target genes of differentially expressed miRNAs was further performed based on the KEGG database to reveal their participation in the molecular mechanisms of the therapeutic effects of noni fruit juice on acute gouty arthritis in mice. The results showed the 44 different KEGG pathways in the six pairwise comparisons of the experimental and control groups in mice were involved in seven categories of KEGG terms, including cellular processes, environmental information processing, genetic information processing, human diseases, metabolism, and organismal systems (Figure 5). The highest numbers of differentially expressed miRNAs (ranging from 5649 to 5696) were enriched in the category of human diseases, followed by the categories of organismal systems (ranging from 4658 to 4709) and metabolism (ranging from 3148 to 3178). The lowest numbers of differentially expressed miRNAs (ranging from 1428 to 1444) were mapped in the category of genetic information processing, while the categories of cellular processes and environmental information processing contained ~2400 (ranging from 2426 to 2441) and ~2800 (ranging from 2823 to 2835) differentially expressed miRNAs, respectively. These results of the KEGG enrichment analysis based on the target genes of the differentially expressed miRNAs indicated that many metabolic pathways were affected in mice treated with noni fruit juice, suggesting that these differentially expressed miRNAs may be involved in the metabolic pathways related to acute gouty arthritis in mice. These results were consistent with the results based on the pathogenetic investigations in our study and with those reported previously [17]. Further studies are required to reveal the explicit functions of these novel miRNAs in the occurrence of acute gouty arthritis and the pharmacological mechanisms of noni fruit juice in the treatment of acute gouty arthritis in mice.

**Figure 6:**
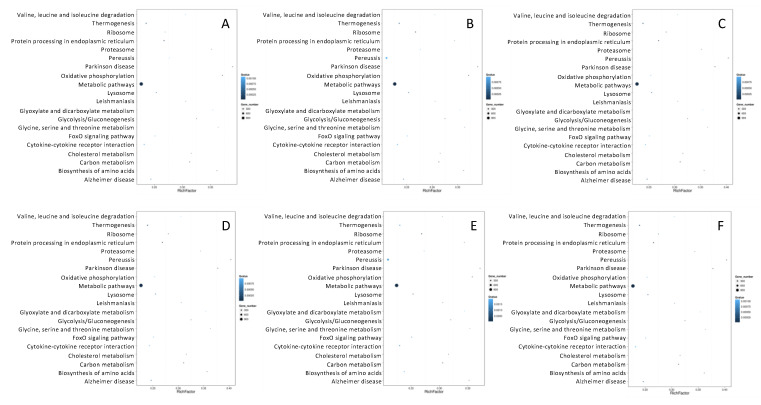
Scatter plots of the top 20 enriched pathway terms based on the Kyoto Encyclopedia of Genes and Genomes (KEGG) database of the differentially expressed microRNAs identified in the six pairwise comparisons of the three experimental and the control groups of mice, including normal control group vs. model group (**A**), normal control group vs. colchicine group (**B**), normal control group vs. noni fruit juice group (**C**), model group vs. noni fruit group (**D**), model group vs. colchicine group (**E**), and noni fruit juice group vs. colchicine group (**F**). A total of over 900 functional genes were annotated in the metabolic pathways among the six pairwise comparisons of the experimental and control groups of mice. The rich factor is the ratio of target gene numbers of the differentially expressed miRNAs annotated in each pathway term to all gene numbers annotated in this pathway term. The greater value of Rich Factor indicates the greater degree of enrichment. The Q-values are corrected *p*-values ranging from 0~1 with the lower Q-value representing the greater level of enrichment.

In the top 20 enriched pathway terms mapped in the KEGG database, over 900 functional genes were annotated in the metabolic pathways among the six pairwise comparisons of the experimental and control groups of mice, while less than 300 genes were mapped on other 19 pathway terms (Figure 6). Future studies are needed to explicitly investigate the functions of these genes, in particular the genes annotated in the metabolic pathways, in the occurrence of acute gouty arthritis and the treatment of noni fruit juice on acute gouty arthritis in mice.

Studies have shown that two miRNAs (i.e., mir-30a-5p and miR-15b-5p) are involved in osteoarthritis by combining with two long noncoding RNAs (i.e., LINC00461 and LINC00662) to increase the inflammation and three types of cytokines (i.e., IL-6, IL-8, and TNF-α), respectively [25,29,81]. These results were consistent with the findings revealed in our study based on the KEGG enrichment analysis, showing that these two miRNAs were responsive to the treatments of noni fruit juice and colchicine with up- and downregulated expressions in model mice and mice treated with both noni fruit juice and colchicine, respectively (Table 5). These results demonstrated the therapeutic effects of noni fruit juice on acute gouty arthritis in mice. Furthermore, studies have shown that other four miRNAs (i.e., miR-122-5p, miR-146a-5p, miR-17-5p, and miR181c-5p) are involved in uric acid nephropathy, acute pneumonia, LPS-induced inflammatory injury, and poststroke inflammation, by combining with long noncoding RNAs of ANRIL, SNHG16, NEAT1, and Malat1, respectively [26,28,33,82]. Similarly, our results showed that these four miRNAs responded to the treatments of noni fruit juice and colchicine, showing up- and downregulated expressions in model mice and mice treated with both noni fruit juice and colchicine, respectively, indicating the therapeutic effects of noni fruit juice on acute gouty arthritis in mice. Further studies are necessary to verify the detailed functions of these miRNAs in the development and treatment of acute gouty arthritis in mice.

Based on the KEGG enrichment analysis, we examined the expression patterns of both known and novel miRNAs identified in our study targeting five types of cytokines, including the two types of cytokines (i.e., the TNF-α and NALP3) examined in our pathogenetic investigations of acute gouty arthritis of this study, involved in the major metabolic pathways related to both acute gouty arthritis, i.e., toll-like receptor 4 (TLR4), NF-kB, and NLRP3, and gouty arthritis, i.e., p53 and forkhead-box class O (FoxO) (Table 6; Table S6). Our results showed that a large number of novel miRNAs were involved in these pathways related to both acute gouty arthritis and gouty arthritis, showing altered levels of accumulation of these cytokines. These results were consistent with those based on the pathogenetic investigations in our study and those reported previously [17]. To date, the understanding of the molecular mechanisms of miRNAs regulating the development of acute gouty arthritis remain unclear [83]. Further studies are necessary to verify the detailed regulatory functions of these novel miRNAs in the occurrence of acute gouty arthritis in mice and to further explore the pharmacological mechanisms of noni fruit juice in the treatment of acute gouty arthritis.

## 4. Conclusions

In this study, we investigated the therapeutic effects of noni fruit juice on treating the acute gouty arthritis in mice induced with MSU. Results showed that in comparison to the model group, the degree of paw swelling in mice was significantly decreased in both the positive control (treated with colchicine) and the noni fruit juice groups, while the contents of two proinflammatory cytokines NALP3 and TNF-α in serum of mice in both the positive control and the noni fruit juice groups were significantly decreased. These results demonstrated the therapeutic effects of noni fruit juice on the treatment of acute gouty arthritis in mice. Furthermore, based on the high-throughput sequencing technology, a group of differentially expressed miRNAs were identified involved in the pathogenetic mechanisms of acute gouty arthritis in mice. Results of the functional enrichment analyses of the target genes of differentially expressed miRNAs based on both GO and KEGG databases revealed the regulatory effects of these miRNAs on the treatment of acute gouty arthritis by noni fruit juice, indicating the potential therapeutic and pharmacological significance of noni fruit juice on the treatment of acute gouty arthritis in mice. These results demonstrate that these miRNAs are involved in the regulation of pathogenetic mechanisms of acute gouty arthritis, providing evidence to support the pharmacological effects of noni fruit juice on the treatment of acute gouty arthritis in mice. It is noted that future studies are necessary to investigate the explicit functions of these miRNAs in the regulation of the pathogenetic mechanisms of and the pharmacological effects of noni fruit juice on the treatment of acute gouty arthritis in mice. Due to their promising therapeutic effects in treatment of acute gouty arthritis, we foresee a prospective future of the agricultural cultivation and nutritional development of noni plants.

## Figures and Tables

**Table 1:** The paw swelling (mm) in mice of the three experimental and the control groups before and after (1 and 5 days) treatments. Data are presented as mean ± standard deviation with the paw swelling degree (i.e., the difference of the paw thickness before and after the treatment) given in parentheses (*n* = 10).

Group of Mice	Day 0	Day 1	Day 5
Normal control group	1.69 ± 0.08	1.72 ± 0.12 (0.12 ± 0.08) **	1.71 ± 0.08 (0.04 ± 0.02) **
Model group	1.70 ± 0.10	3.70 ± 0.25 (2.01 ± 0.22)	2.89 ± 0.27 (1.19 ± 0.24)
Colchicine group	1.70 ± 0.11	3.50 ± 0.21 (1.80 ± 0.16) *	2.31 ± 0.25 (0.61 ± 0.27) **
Noni fruit juice group	1.74 ± 0.07	3.65 ± 0.16 (1.91 ± 0.16)	2.68 ± 0.20 (0.94 ± 0.21) *

Symbols “*” and “**” indicate the significant difference at *p* < 0.05 and *p* < 0.01, respectively, in comparison to the model group.

**Table 2:** Contents of NALP3 and TNF-α detected in the serum of three experimental and the control groups of mice 5 days after treatments. Data are presented as mean ± standard deviation (*n* = 10). Symbols “*” and “**” indicate the significant difference at *p* < 0.05 and *p* < 0. 01, respectively, in comparison to the model group.

Group of Mice	NALP3 (pg/mL)	TNF-α (pg/mL)
Normal control group	86.09 ± 6.92 **	37.07 ± 4.49 **
Model group	128.90 ± 9.31	71.94 ± 9.76
Colchicine group	102.73 ± 7.25 **	43.09 ± 3.76 **
Noni fruit juice group	110.19 ± 8.25 *	62.06 ± 7.29 *

**Table 3:** Statistics of small RNA sequencing in three experimental and the control groups of mice based on BGISEQ-500 technology. The percentage of clean tag is calculated as (clean tag counts/raw tag counts) × 100%. The percentage of mapped tag is calculated as (mapped tag counts/clean tag counts) × 100%.

Sample	Raw Tag	Clean Tag (%)	Q20 of Clean Tag (%)	Mapped Tag (%)
Normal control group	29,268,292	23,957,783 (81.86)	99.20	21,749,074 (90.78)
Model group	26,562,125	18,683,543 (70.34)	99.30	15,904,741 (85.13)
Colchicine group	27,814,896	25,478,925 (91.60)	99.20	24,180,100 (94.90)
Noni fruit juice group	28,571,428	24,142,747 (84.50)	99.30	22,911,510 (94.90)

**Table 4:** Expression patterns of microRNAs targeting the genes involved in the formation of NLRP3 (NALP3) inflammasome complex and the positive and negative regulations of NLRP3 inflammasome complex assembly based on the Gene Ontology (GO) annotation in the six pairwise comparisons among the experimental and control groups of mice. Data are presented as number of (known)/(novel) miRNAs. Symbols “↑” and “↓” indicate up- and down-regulation, respectively.

GO Term and Gene	Control vs. Model Groups	Control vs. Noni Fruit Juice Groups	Control vs. Colchicine Groups	Model vs. Noni Fruit Juice Groups	Noni Fruit Juice vs. Colchicine Groups	Model vs. Colchicine Groups
Formation of NLRP3 inflammasome complex (GO: 0072559)						
NM_026960.4	(2↑)/(9↑, 13↓)	(3↓)/(21↑, 8↓)	(3↓)/(2↑, 17↓)	(3↓)/(26↑, 8↓)	(1↑)/(28↓)	(3↓)/(2↑, 11↓)
NM_009807.2	(0) /(1↑, 1↓)	(0)/(5↑)	(0)/(1↓)	(0)/(6↑, 1↓)	(0)/(6↓)	(0)/(1↓)
XM_006532857.1	(2↑, 1↓)/(36↑, 95↓)	(2↓)/(117↑, 53↓)	(5↓)/(10↑, 115↓)	(1↑, 3↓)/(147↑, 27↓)	(4↓)/(13↑, 174↓)	(4↓)/(17↑, 63↓)
Positive regulation of NLRP3 inflammasome complex assembly (GO: 1900226)						
XM_006503857.2	(0)/(5↑, 3↓)	(0)/(9↑, 2↓)	(0)/(1↑, 8↓)	(0)/(10↑, 4↓)	(0)/(14↓)	(0)/(7↓)
XM_006535624.3	(1↑, 1↓)/(16↑, 21↓)	(1↑, 1↓)/(41↑, 12↓)	(1↓)/(27↓)	(0)/(44↑, 12↓)	(1↓)/(1↑, 57↓)	(1↓)/(4↑, 20↓)
NM_153564.2	(0)/(3↑, 13↓)	(1↑)/(21↑, 6↓)	(0)/(15↓)	(1↑, 1↓)/(28↑, 3↓)	(1↓)/(1↑, 27↓)	(1↓)/(2↑, 5↓)
NM_021297.3	(5↑)/(72↑, 125↓)	(3↑, 3↓)/(193↑, 71↓)	(5↓)/(8↑, 139↓)	(1↑, 4↓)/(226↑, 57↓)	(1↑, 4↓)/(14↑, 253↓)	(6↓)/(23↑, 86↓)
Negative regulation of NLRP3 inflammasome complex assembly (GO: 1900227)						
NM_022432.4	(3↑, 2↓)/(54↑, 99↓)	(1↓)/(143↑, 59↓)	(3↓)/(9↑, 132↓)	(1↑, 3↓)/(170↑, 39↓)	(1↓)/(11↑, 200↓)	(3↓)/(14↑, 78↓)
NM_019453.2	(5↑, 1↓)/(45↑, 73↓)	(2↑)/(115↑, 46↓)	(5↓)/(10↑, 88↓)	(1↑, 4↓)/(124↑, 35↓)	(4↓)/(11↑, 148↓)	(6↓)/(15↑, 59↓)

**Table 5:** Expression patterns of known microRNAs involved in various types of inflammation based on the Kyoto Encyclopedia of Genes and Genomes (KEGG) enrichment of the six pairwise comparisons of the three experimental and the control groups of mice. Symbols “↑” and “↓” indicate up- and downregulation, respectively.

Medical Condition	Control vs. Model Groups	Control vs. Noni Fruit Juice Groups	Control vs. Colchicine Groups	Model vs. Noni Fruit Juice Groups	Model vs. Colchicine Groups	Noni Fruit Juice vs. Colchicine Groups
Osteoarthritis	miR-30a-5p ↑		miR-30a-5p ↓	miR-30a-5p ↓	miR-30a-5p ↓	miR-30a-5p ↓
Osteoarthritis	miR-15b-5p ↑	miR-15b-5p ↑	miR-15b-5p ↓		miR-15b-5p ↓	miR-15b-5p ↓
Uric acid nephropathy	miR-122-5p ↑	miR-122-5p ↑	miR-122-5p ↑			miR-122-5p ↓
Acute pneumonia		miR-146a-5p ↑				miR-146a-5p ↓
LPS-induced inflammatory injury		miR-17-5p ↓	miR-17-5p ↓	miR-17-5p ↓	miR-17-5p ↓	miR-17-5p ↓
Poststroke inflammation		miR-181c-5p ↓	miR-181c-5p ↓	miR-181c-5p ↓	miR-181c-5p ↓	

**Table 6:** Expression patterns of microRNAs in the pairwise comparisons among the three experimental and the control groups of mice targeting the five main proinflammatory cytokines involved in the major metabolic pathways related to acute gouty arthritis based on the Kyoto Encyclopedia of Genes and Genomes (KEGG) enrichment. Data are presented as number of (known)/(novel) miRNAs. Symbols “↑” and “↓” indicate up- and downregulation, respectively.

Cytokine	Control vs. Model Groups	Control vs. Noni Fruit Juice Groups	Control vs. Colchicine Groups	Model vs. Noni Fruit Juice Groups	Noni Fruit Juice vs. Colchicine Groups	Model vs. Colchicine Groups
Interleukin-6 (IL-6)	(2↑)/(4↑, 2↓)	(2↓)/(9↑, 1↓)	(2↓)/(1↑, 4↓)	(3↓)/(9↑, 4↓)	(1↑)/(11↓)	(3↓)/(4↓)
TNF-α	(4↑)/(52↑, 89↓)	(3↓)/(98↑, 354↓)	(4↓)/(3↑, 119↓)	(5↓)/(116↑, 43↓)	(2↓)/(5↑, 150↓)	(5↓)/(12↑, 81↓)
NALP3	(2↑, 1↓)/(37↑, 99↓)	(2↓)/(118↑, 57↓)	(5↓)/(10↑, 120↓)	(1↑, 3↓)/(149↑, 27↓)	(4↓)/(13↑, 177↓)	(4↓)/(17↑, 64↓)
Interleukin-1β (IL-1β)	(3↑, 1↓)/(18↑, 39↓)	(1↑)/(49↑, 21↓)	(2↓)/(2↑, 41↓)	(1↑, 3↓)/(57↑, 11↓)	(2↓)/(3↑, 65↓)	(3↓)/(8↑, 21↓)
Monocyte chemoattractant protein-1 (MCP-1)	(1↑)/(11↑, 18↓)	(1↓)/(27↑, 10↓)	(1↓)/(1↑, 20↓)	(2↓)/(31↑, 8↓)	(0)/(34↓)	(2↓)/(11↓)

## Data Availability

The data presented in this study are available on request from the corresponding authors.

## Supplementary Materials

The following are available online at https://www.mdpi.com/article/10.3390/foods10071638/s1, Figure S1: Proportions of different types of small RNAs identified in the normal control, the model, the noni fruit juice, and the positive control (treated with colchicine) groups of mice. Figure S2: Heatmaps of hierarchical clustering based on the intersection (left) and union (right) of differentially expressed microRNAs in the model, the noni fruit juice, the positive control (treated with colchicine), and the normal control groups of mice. Figure S3: Heatmaps of hierarchical clustering based on the intersection (left) and union (right) of differentially expressed microRNAs in six pairwise comparisons of three experimental and the control groups of mice. Table S1: Lists of different types of small RNAs identified in the normal control, the model, the noni fruit juice, and the positive control (treated with colchicine) groups of mice. Table S2: Lists of predicted known and novel microRNAs in the normal control, the model, the positive control (treated with colchicine), and the noni fruit juice groups of mice. Table S3: List of differentially expressed microRNAs based on the six pairwise comparisons of the normal control, the model, the positive control (treated with colchicine), and the noni fruit juice groups of mice. Table S4: Target genes identified by both TargetScan and miRanda of the microRNAs of the model, the positive control (treated with colchicine), the noni fruit juice, and the normal control groups of mice. Table S5: List and expression patterns of microRNAs with the target genes involved in the formation of NLRP3 (NALP3) inflammasome complex and the positive and negative regulations of NLRP3 inflammasome complex assembly based on the Gene Ontology (GO) annotation in the six pairwise comparisons of the normal control, the model, the positive control (treated with colchicine), and the noni fruit juice groups of mice. Table S6: List and expression patterns of both known and novel microRNAs targeting five types of proinflammatory cytokines involved in the major metabolic pathways related to both acute gouty arthritis and gouty arthritis based on the Kyoto Encyclopedia of Genes and Genomes (KEGG) enrichment in the six pairwise comparison of the normal control, the model, the positive control (treated with colchicine), and the noni fruit juice groups of mice.
