# Supplementary material for: MicroRNAs Involved in the Therapeutic Functions of Noni (Morinda citrifolia L.) Fruit Juice in the Treatment of Acute Gouty Arthritis in Mice Induced with Monosodium Urate
Source: Foods. 2021 Jul 15;10(7):1638. doi: 10.3390/foods10071638 (PMC8308103; doi:10.3390/foods10071638)
Supplement: Supplementary file 1 [file foods-10-01638-s001.zip › Figure S1.pdf]

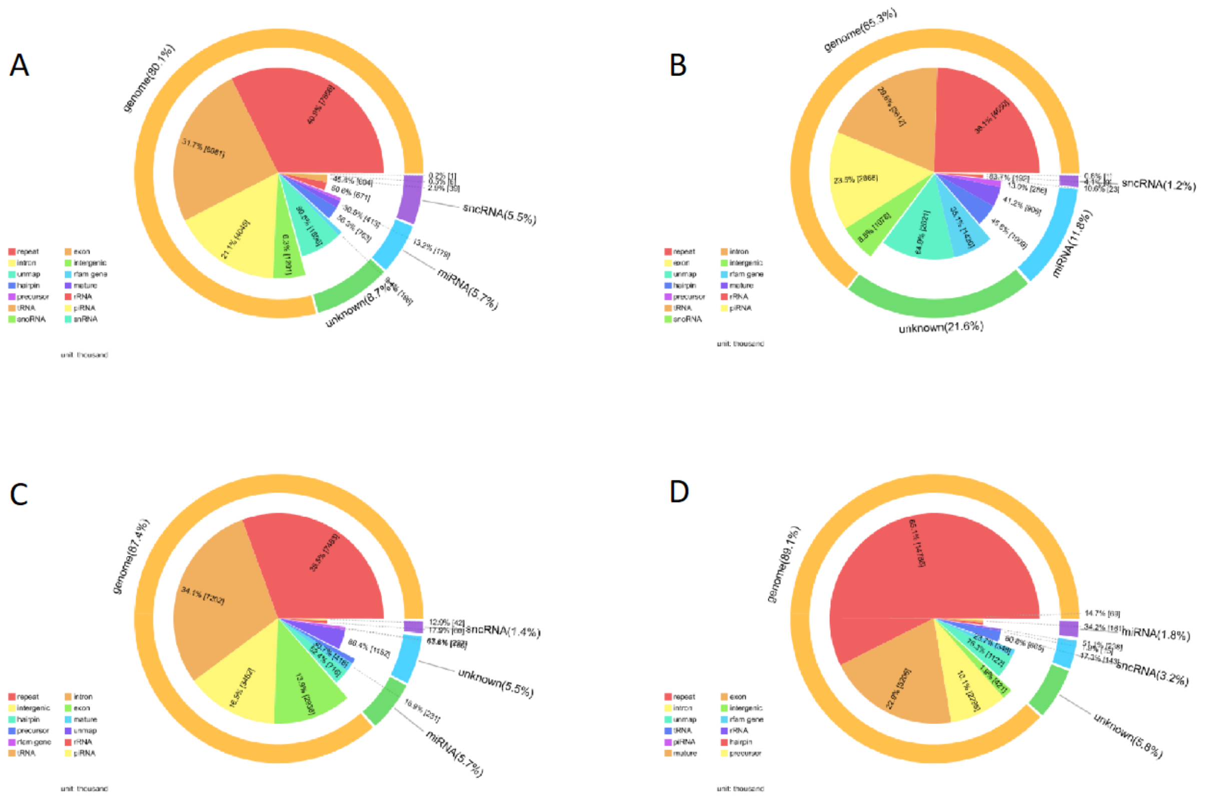

Figure S1. Proportions of different types of small RNAs identified in the normal control group (A), the model group (B), the noni fruit juice group (C), and the colchicine group (D) of mice. In order to obtain each small RNA corresponding to only one annotation, the priority rule of microRNA > piwi-interacting RNA (piRNA) > small nucleolar RNA (snoRNA) > Rfam > other small RNAs.
