# Supplementary material for: MicroRNAs Involved in the Therapeutic Functions of Noni (Morinda citrifolia L.) Fruit Juice in the Treatment of Acute Gouty Arthritis in Mice Induced with Monosodium Urate
Source: Foods. 2021 Jul 15;10(7):1638. doi: 10.3390/foods10071638 (PMC8308103; doi:10.3390/foods10071638)
Supplement: Supplementary file 1 [file foods-10-01638-s001.zip › Figure S2.pdf]

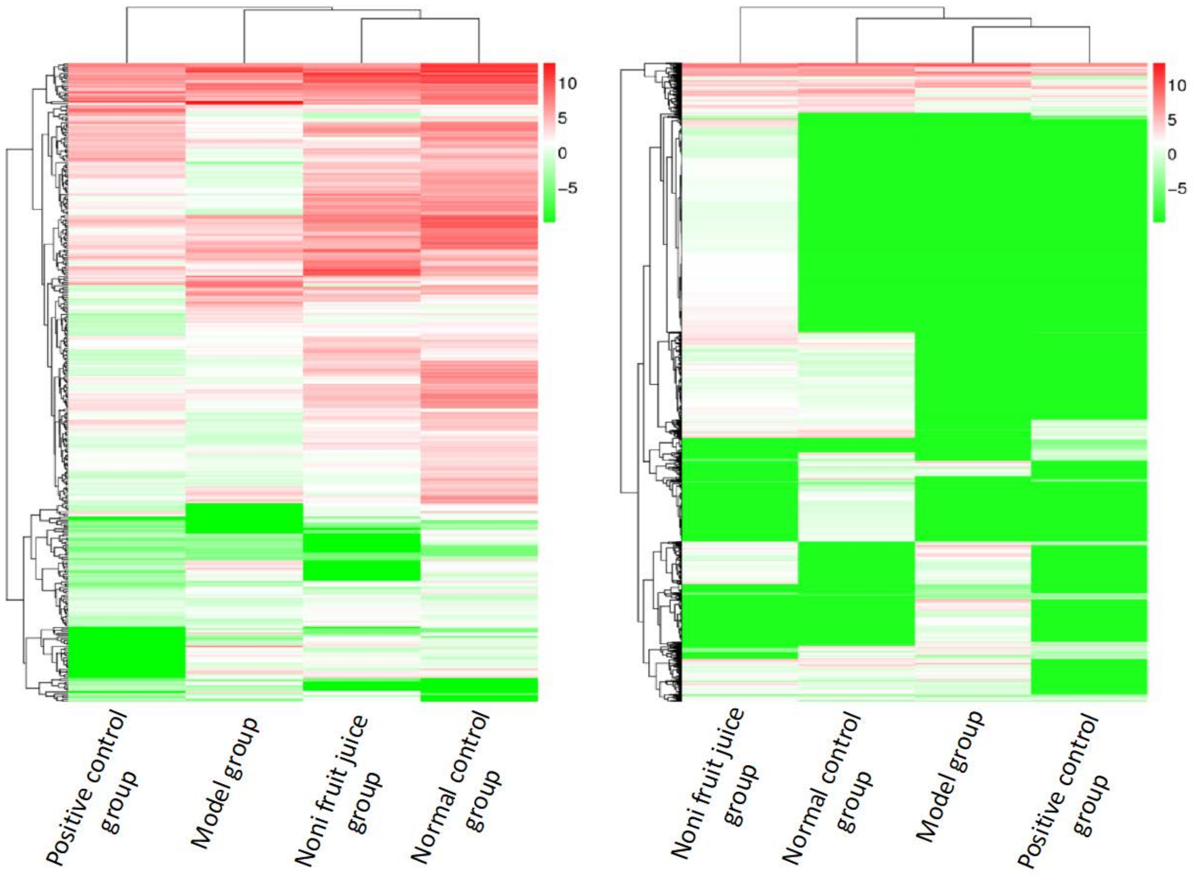

Figure S2. Heatmaps of hierarchical clustering based on the intersection (left) and union (right) of differentially expressed microRNAs in the model group, the noni fruit juice group, the colchicine group (i.e., the positive control), and the normal control group of mice. Folder changes are indicated by the red bars showing up-regulation and green bars showing down-regulation.
