# Supplementary material for: MicroRNAs Involved in the Therapeutic Functions of Noni (Morinda citrifolia L.) Fruit Juice in the Treatment of Acute Gouty Arthritis in Mice Induced with Monosodium Urate
Source: Foods. 2021 Jul 15;10(7):1638. doi: 10.3390/foods10071638 (PMC8308103; doi:10.3390/foods10071638)
Supplement: Supplementary file 1 [file foods-10-01638-s001.zip › Figure S3.pdf]

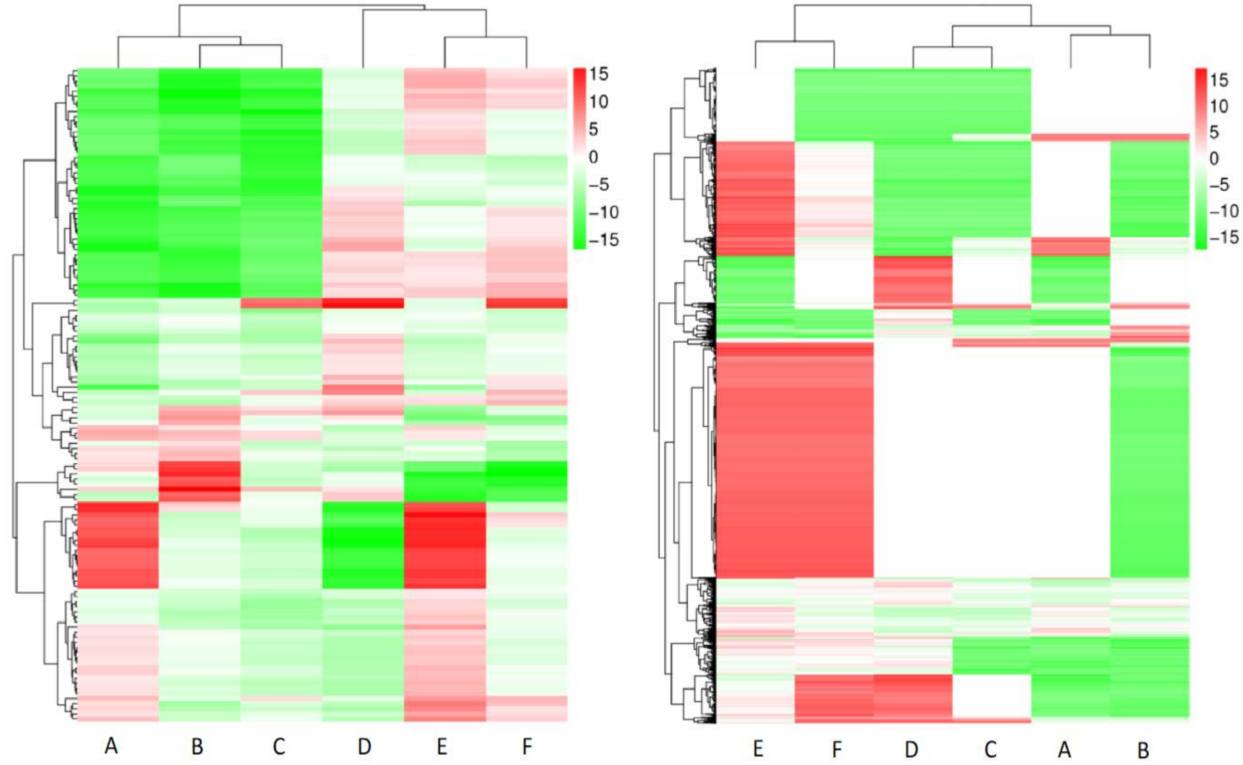

Figure S3. Heatmaps of hierarchical clustering based on the intersection (left) and union (right) of differentially expressed microRNAs in six pairwise comparisons of four groups of mice, including model group vs. positive control group (A), noni fruit juice group vs. positive control group (B), normal control group vs. positive control group (C), normal control group vs. model group (D), model group vs. noni fruit juice group (E), and normal control group vs. noni fruit juice group (F). The positive control group is treated with colchicine. Folder changes are indicated by the red bars showing up-regulation and green bars showing down-regulation.
